# How mNGS transforms care for non-verbal elderly stroke patients with pneumonia

**DOI:** 10.3389/fcimb.2026.1814320

**Published:** 2026-06-23

**Authors:** Yuhai Dang

**Affiliations:** Department of Respiratory and Critical Care Medicine, Guangxi Jiangbin Hospital, Nanning, Guangxi, China

**Keywords:** antimicrobial stewardship, elderly, metagenomic next-generation sequencing, mortality, non-verbal, pathogen detection, stroke-associated pneumonia

## Abstract

**Background:**

Stroke-associated pneumonia (SAP) is a severe complication in non-verbal elderly stroke patients, with diagnosis hindered by the low sensitivity and slow turnaround of conventional microbial culture.

**Methods:**

A single-center retrospective cohort study was conducted on 64 non-verbal elderly SAP patients (≥65 years) admitted to Guangxi Jiangbin Hospital from 2018 to 2022, divided into an mNGS group (n=30, sputum/BALF tested by metagenomic next-generation sequencing) and a control group (n=34, conventional culture). Propensity score matching (1:1) was used to balance baseline characteristics, and clinical outcomes and pathogen detection efficiency were compared between groups. Multivariable Cox regression adjusted for hypoalbuminemia, electrolyte disturbance and stroke severity.

**Results:**

mNGS detected more bacterial pathogens (37 vs.27 in sputum, 37 vs.21 in BALF) and identified 3 viral and 2 atypical pathogens undetectable by culture, with a negative rate of 13.3% (vs.20.0% for sputum culture, 43.3% for BALF culture). 73.3% of mNGS group patients received antimicrobial therapy adjustment. After adjustment, the mNGS group had notably higher 28-day (96.7% vs.76.5%; adjusted HR = 0.32, *P* = 0.032) and 90-day survival (76.7% vs.44.1%; adjusted HR = 0.41, *P* = 0.024), lower invasive mechanical ventilation rate (40.0% vs.64.7%, *P* = 0.048), shorter median antibiotic duration (14 vs.21 days, *P* = 0.016) and lower median hospitalization costs (¥32,450 vs.¥89,310, *P* < 0.001).

**Conclusion:**

mNGS enables more comprehensive pathogen detection in non-verbal elderly SAP patients, guides targeted antimicrobial therapy, and is associated with improved survival and reduced healthcare resource consumption. However, large-sample multicenter prospective studies are needed to validate these findings due to the study’s limitations.

## 1 Introduction

Stroke-associated pneumonia (SAP) is a prevalent and life-threatening complication of acute ischemic or hemorrhagic stroke, particularly in the elderly population, with profound impacts on patient prognosis and healthcare systems (Hannawi et al., 2013). As a leading cause of increased mortality, functional decline and prolonged hospitalization in stroke survivors (Hannawi et al., 2013; Cieplik et al., 2020), SAP is closely linked to aspiration caused by impaired consciousness, dysphagia and reduced airway clearance in stroke patients (Hannawi et al., 2013; de Mello et al., 2025). The non-verbal subgroup (with Glasgow Coma Scale verbal score 1–2 or severe aphasia/dysarthria) faces greater diagnostic challenges, as they cannot report respiratory symptoms, leading to delayed identification of infection and suboptimal treatment.

Timely and accurate pathogen identification is the cornerstone of effective SAP management, as it guides targeted antimicrobial therapy and reduces the risk of antibiotic resistance (Yang et al., 2022; Fishman, 2006; Allerberger and Mittermayer, 2008). However, conventional microbiological culture—the gold standard for pathogen detection—has inherent limitations: a 48–72 hour turnaround time for preliminary results, low sensitivity for fastidious bacteria, atypical pathogens and viruses, and a high negative detection rate in clinical practice (Vepřeková and Pokorná, 2013; Fishman, 2019). These deficiencies result in empirical broad-spectrum antibiotic use in the critical early phase after stroke, which may be inappropriate, overly broad or insufficient, further exacerbating clinical deterioration and antimicrobial resistance (Fishman, 2006; Allerberger and Mittermayer, 2008).

Metagenomic next-generation sequencing (mNGS) has emerged as a transformative diagnostic tool in clinical microbiology, offering an unbiased, culture-independent approach to detect all nucleic acids in clinical samples (Fishman, 2019). Capable of identifying bacteria, fungi, viruses and parasites in a single assay within 24–48 hours, mNGS has demonstrated higher diagnostic yield in severe infections such as pneumonia, meningitis and sepsis, especially for difficult-to-culture pathogens and polymicrobial infections (Ata et al., 2020; Fishman, 2019; Yarahuan et al., 2023). Despite these technical advantages, the clinical utility and real-world impact of mNGS in SAP—particularly in non-verbal elderly patients with unique diagnostic and therapeutic needs—remain underexplored (Rodriguez-Guerra et al., 2021; Yarahuan et al., 2023). Critical questions persist regarding whether mNGS-guided diagnosis translates to improved survival, reduced antibiotic exposure and lower healthcare costs in this vulnerable cohort (Fishman, 2006; Hannawi et al., 2013; Cieplik et al., 2020).

To address this research gap, we conducted a retrospective cohort study comparing pathogen detection efficiency and clinical outcomes between non-verbal elderly SAP patients diagnosed by mNGS and conventional culture (Ata et al., 2020). Propensity score matching was used to minimize confounding by baseline severity and comorbidities, providing preliminary evidence for the potential value of mNGS in the diagnostic pathway of SAP. This study aimed to evaluate whether mNGS integration improves survival rates, optimizes antimicrobial stewardship (Yang et al., 2022), reduces invasive respiratory support needs and lowers hospitalization costs, to inform clinical decision-making for this high-risk population.

## 2 Materials and methods

### 2.1 Study design and population

This single-center retrospective cohort study was conducted at the Department of Respiratory and Critical Care Medicine, Guangxi Jiangbin Hospital, from January 2018 to December 2022. The study protocol was approved by the hospital’s Ethics Committee (Approval No.LW2025-021), and informed consent was waived due to the retrospective design and use of anonymized clinical data, in accordance with the Declaration of Helsinki.

#### 2.1.1 Inclusion criteria

(1) Age ≥65 years; (2) Diagnosis of SAP per the *Chinese Expert Consensus on the Diagnosis and Treatment of Stroke-Associated Pneumonia (2019 Update Edition)*, defined as pneumonia occurring within 7 days of acute stroke onset with consistent clinical and radiographic findings; (3) Non-verbal status (Glasgow Coma Scale [GCS] verbal score 1–2 or documented aphasia/severe dysarthria precluding meaningful communication).

#### 2.1.2 Exclusion criteria

(1) Active malignant tumor; (2) Receipt of immunosuppressive therapy or HIV infection; (3) Incomplete medical records or loss to 90-day follow-up.

A total of 64 patients meeting the criteria were enrolled and divided into two groups based on pathogen detection method: the mNGS group (n=30) received sputum and/or bronchoalveolar lavage fluid (BALF) testing by mNGS; the control group (n=34) underwent only conventional sputum/BALF culture. A CONSORT-style flowchart was used to document patient screening, exclusion and final allocation.

### 2.2 Diagnostic criteria for SAP

SAP diagnosis required concurrent clinical and radiographic criteria (Hannawi et al., 2013):

- Clinical: Fever ≥38 °C, leukopenia (WBC ≤4×10^9^/L) or leukocytosis (WBC ≥10×10^9^/L), altered mental status (in patients ≥70 years), plus at least two of: purulent sputum, increased airway secretions, cough, dyspnea/tachypnea (>25 breaths/min), abnormal pulmonary auscultation, or worsening gas exchange (PaO_2_/FiO_2_ ≤300).
- Radiographic: New or progressive pulmonary infiltrate, consolidation, or ground-glass opacity on chest computed tomography or X-ray.

### 2.3 mNGS and conventional microbiological detection

mNGS detection: Sputum/BALF samples were sent to a third-party commercial laboratory (Guangzhou KingMed Diagnostics Group Co., Ltd., Guangzhou, China) for mNGS testing. This laboratory holds clinical laboratory accreditation under the Clinical Laboratory Improvement Amendments (CLIA) and is certified by the College of American Pathologists (CAP). The laboratory’s quality management system complies with ISO 15189:2012 standards. All mNGS procedures, including nucleic acid extraction, library construction, sequencing, and bioinformatic analysis, were performed according to the laboratory’s standard operating protocols validated for clinical respiratory specimens.

Conventional culture: Sputum/BALF samples were processed per hospital standard operating procedures for aerobic, anaerobic and fungal culture. Strain identification and antimicrobial susceptibility testing were performed using the VITEK 2 system (bioMérieux, France).

### 2.4 Data collection

Electronic medical records were extracted to collect the following variables:

#### 2.4.1 Demographic and baseline characteristics

Age, gender, stroke type, National Institutes of Health Stroke Scale (NIHSS) score, GCS score, comorbidities (hypertension, diabetes, coronary heart disease, liver/renal insufficiency, gastrointestinal bleeding);

#### 2.4.2 Complications

Hypoalbuminemia (serum albumin <35 g/L), electrolyte disturbance (serum Na <135 mmol/L or >145 mmol/L, and/or K <3.5 mmol/L or >5.5 mmol/L);

#### 2.4.3 Pathogen detection

Types/quantities of pathogens identified by sputum culture, BALF culture and mNGS, negative detection rate, consistency between methods;

#### 2.4.4 Treatment details

Antimicrobial type/duration, tracheal intubation, invasive mechanical ventilation use;

#### 2.4.5 Outcome indicators

28-day/90-day all-cause survival/mortality, total hospitalization costs (raw data, unadjusted for inflation).

### 2.5 Timeline of diagnosis, antibiotic initiation, and mNGS implementation

To contextualize the clinical utility of mNGS, the following time intervals were extracted from medical records for each patient in the mNGS group:

1. time from SAP diagnosis to first antibiotic administration (hours);
2. time from SAP diagnosis to sputum/BALF sampling for conventional culture;
3. time from SAP diagnosis to mNGS sample collection;
4. time from mNGS sample collection to result reporting (days);
5. time from mNGS result reporting to any antimicrobial therapy adjustment (de-escalation or escalation), recorded as “time to adjustment”.

These intervals are reported as median (IQR) in Results and used to interpret the clinical feasibility of mNGS-guided therapy in non-verbal elderly patients.

### 2.6 Statistical analysis

SPSS 26.0 and R 4.2.0 software were used for all statistical analyzes, with a two-sided *P* < 0.05 considered statistically significant.

- Descriptive statistics: Continuous variables with non-normal distribution (consistent with clinical data characteristics) were expressed as median (interquartile range, IQR); categorical variables as frequency (percentage, n, %).
- Group comparison: Mann–Whitney U test for continuous variables; Chi-square test or Fisher’s exact test for categorical variables (when theoretical frequency of any cell <5).
- Propensity score matching (PSM): 1:1 PSM with a caliper of 0.2 was used to balance baseline characteristics (age, gender, NIHSS, comorbidities) between groups and reduce selection bias.
- Survival analysis: Kaplan–Meier curves were used to plot survival rates, and multivariable Cox regression was performed to calculate adjusted hazard ratios (HR) and 95% confidence intervals (CI), adjusting for hypoalbuminemia, electrolyte disturbance and stroke severity (NIHSS score).
- Consistency analysis: Kappa consistency test was used to evaluate the agreement between mNGS and conventional culture (Kappa <0.40: poor; 0.40–0.75: moderate; >0.75: good).

## 3 Results

### 3.1 Baseline characteristics

After 1:1 PSM, 26 matched pairs (52 patients) were included in the balanced baseline analysis, with no significant differences in age, gender, NIHSS score, GCS verbal score or most comorbidities between the mNGS and control groups (*P*>0.05). Hypoalbuminemia and electrolyte disturbance—imbalance in the original cohort—were well-matched after PSM and included as confounding factors in subsequent multivariable analysis. The overall study cohort had a median age of 82 years, with 68% male patients, and all were non-verbal (GCS verbal score 1–2). Baseline characteristics of the original and matched cohorts are shown in **Table 1**.

**Table 1:** Baseline characteristics of the study population (original and matched cohorts).

Characteristic	Original cohort		Matched cohort (PSM 1:1)	
	mNGS (n=30)	Control (n=34)	mNGS (n=26)	Control (n=26)
Age, years, median (IQR)	80.47 ± 1.20	82.71 ± 1.34	81 (76–85)	83 (78–87)
Male, n (%)	21 (70.0)	25 (73.5)	18 (69.2)	19 (73.1)
NIHSS, median (IQR)	13 (9–17)	14 (10–18)	14 (10–18)	15 (11–19)
GCS verbal score 1–2, n (%)	30 (100)	34 (100)	26 (100)	26 (100)
Hypoalbuminemia, n (%)	14 (46.7)	4 (11.8)*	10 (38.5)	9 (34.6)
Electrolyte disturbance, n (%)	11 (36.7)	2 (5.9)*	8 (30.8)	7 (26.9)

*P<0.001 vs. mNGS group (original cohort); P>0.05 for all indicators in matched cohort.

### 3.2 Pathogen detection efficiency

#### 3.2.1 Pathogen spectrum and detection rate

mNGS exhibited higher pathogen detection capacity compared to conventional culture (**Table 2**). In the mNGS group, mNGS identified 37 bacterial strains, 7 fungal strains, 3 viral strains (cytomegalovirus, human herpesvirus I type, Kaposi’s sarcoma virus) and 2 atypical pathogen strains (Mycobacterium tuberculosis complex, Nocardia spp.), while conventional culture failed to detect any viruses or atypical pathogens. For bacterial pathogens, mNGS detected 37 strains (sputum/BALF combined), significantly more than sputum culture (27 strains) and BALF culture (21 strains), with mNGS/sputum and mNGS/BALF detection ratios of 1.37 and 1.76, respectively. Notably, mNGS had a lower fungal detection rate (7 strains) than culture (8 strains).

**Table 2:** Pathogen detection results in the mNGS group (n=30).

Pathogen type	Sputum culture	Balf culture	mNGS	mNGS/sputum ratio	mNGS/BALF ratio	Negative rate (%)
Bacteria	27	21	37	1.37	1.76	—
Fungi	8	8	7	0.875	0.875	—
Virus	0	0	3	—	—	—
Atypical Pathogens	0	0	2	—	—	—
Total Negative	6	13	4	—	—	20.0/43.3/13.3

#### 3.2.2 Negative detection rate

mNGS had the lowest negative detection rate at 13.3% (4/30), significantly lower than sputum culture (20.0%, 6/30) and BALF culture (43.3%, 13/30), indicating higher diagnostic sensitivity for SAP pathogens.

#### 3.2.3 Consistency analysis

mNGS and conventional culture had a 70% overall consistency rate. Kappa consistency test showed moderate consistency between mNGS and sputum culture (Kappa=0.42, 95%CI:0.21–0.63, *P* = 0.03) and poor consistency between mNGS and BALF culture (Kappa=0.38, 95%CI:0.15–0.61, *P* = 0.14).

#### 3.2.4 Timeline of diagnostic and therapeutic interventions (mNGS group only)

The median time from SAP diagnosis to antibiotic initiation was 3 hours (IQR: 2–5). mNGS samples were collected at a median of 2 days (IQR: 1–4) after diagnosis. The median turnaround time from mNGS sample collection to result reporting was 32 hours (IQR: 28–38). Among the 22 patients who received therapy adjustment, the median time from mNGS result reporting to adjustment was 6 hours (IQR: 4–10).

### 3.3 Antimicrobial therapy adjustment

mNGS results guided antimicrobial therapy modification in 22/30 (73.3%) patients in the mNGS group. Of these adjustments, 12 cases (54.5%) were antibiotic de-escalation (discontinuation of unnecessary broad-spectrum antibiotics, reduction of drug spectrum or dosage), and 10 cases (45.5%) were targeted escalation (initiation of antiviral, antimycobacterial or antifungal therapy based on mNGS-detected pathogens missed by culture). No significant therapy adjustment was performed in the control group due to low culture positive rates and lack of pathogen-specific findings.

### 3.4 Clinical outcomes

#### 3.4.1 Survival and mortality

After multivariable Cox regression adjustment for hypoalbuminemia, electrolyte disturbance and NIHSS score, the mNGS group had significantly higher 28-day and 90-day survival rates, and lower all-cause mortality, compared to the control group (**Table 3**). The 28-day survival rate was 96.7% (29/30) in the mNGS group vs.76.5% (26/34) in the control group (adjusted HR = 0.32, 95%CI:0.11–0.91, *P* = 0.032); the 90-day survival rate was 76.7% (23/30) vs.44.1% (15/34) (adjusted HR = 0.41, 95%CI:0.19–0.89, *P* = 0.024). The 28-day and 90-day mortality rates in the mNGS group were 3.3% and 23.3%, respectively, significantly lower than the control group’s 23.5% and 55.9%.

**Table 3:** Clinical outcomes between the mNGS group and control group.

Outcome indicator	mNGS group (n=30)	Control group (n=34)	Adjusted HR/OR (95%CI)	*P* value
28-day survival, n (%)	29 (96.7)	26 (76.5)	0.32 (0.11–0.91)	0.032
90-day survival, n (%)	23 (76.7)	15 (44.1)	0.41 (0.19–0.89)	0.024
Invasive mechanical ventilation, n (%)	12 (40.0)	22 (64.7)	0.38 (0.15–0.96)	0.048
Antibiotic duration, days, median (IQR)	14 (9–22)	21 (14–30)	—	0.016
Hospitalization costs, ¥, median (IQR)	32,450 (21,880–45,600)	89,310 (58,440–124,500)	—	<0.001

#### 3.4.2 Respiratory support needs

There was no significant difference in tracheal intubation rate between the two groups (53.3% vs.55.9%, *P* = 0.838). However, the invasive mechanical ventilation rate in the mNGS group was 40.0% (12/30), significantly lower than the control group’s 64.7% (22/34) (adjusted OR = 0.38, 95%CI:0.15–0.96, *P* = 0.048), indicating a lower risk of severe respiratory failure in the mNGS group.

#### 3.4.3 Antibiotic duration and hospitalization costs

The mNGS group had a significantly shorter median antibiotic treatment duration (14 days, IQR:9–22) than the control group (21 days, IQR:14–30) (*P* = 0.016). Median total hospitalization costs in the mNGS group were ¥32,450 (IQR:21,880–45,600), approximately one-third of the control group’s ¥89,310 (IQR:58,440–124,500), with a statistically significant difference (*P* < 0.001).

#### 3.4.4 Cost breakdown and estimated net savings

To clarify the drivers of lower total hospitalization costs in the mNGS group, we performed a descriptive cost decomposition based on hospital billing records. The median total cost in the control group (¥89,310) was primarily composed of: prolonged antibiotic therapy (estimated 21 vs.14 days, contributing to drug acquisition and administration costs), higher frequency of invasive mechanical ventilation (64.7% vs.40.0%, incurring ICU bed, ventilator, and daily management fees), and extended length of stay (not directly shown but reflected in cost differences). In contrast, the mNGS group incurred an additional upfront cost for mNGS testing (¥2,500–3,500 per sample, including equipment amortization, reagents, and labor), which was offset by shorter antibiotic duration and lower ventilation-associated expenses. The estimated net saving associated with the mNGS-guided strategy was approximately ¥56,860 per patient (median total cost difference), although this figure does not account for regional price variations or inflation.

## 4 Discussion

This study is to focus on the clinical value of mNGS in non-verbal elderly SAP patients—a high-risk subgroup with significant diagnostic barriers—and confirms that mNGS outperforms conventional culture in pathogen detection, and its application is associated with improved survival outcomes and optimized healthcare resource utilization. The key findings of this study, their clinical implications, and limitations are discussed below, in the context of existing literature (Fishman, 2006; Hannawi et al., 2013; Fishman, 2019; Cieplik et al., 2020).

### 4.1 mNGS enhances pathogen detection in SAP

Conventional culture’s low sensitivity and narrow pathogen spectrum are major bottlenecks in SAP diagnosis (Vepřeková and Pokorná, 2013; Fishman, 2019), and this limitation is more pronounced in non-verbal patients where clinical symptom assessment is unavailable. Our results show that mNGS not only detects 37% more bacterial pathogens than sputum culture but also identifies viruses and atypical pathogens (CMV, Mycobacterium tuberculosis, Nocardia) that are completely missed by culture. These pathogens are important causes of severe SAP in the elderly (de Mello et al., 2025), and their undetection by culture leads to ineffective empirical therapy and poor prognosis. Additionally, mNGS’s negative detection rate (13.3%) is far lower than that of BALF culture (43.3%), which is critical for reducing unnecessary antibiotic use in culture-negative patients.

The moderate consistency between mNGS and sputum culture (Kappa=0.42) and poor consistency with BALF culture (Kappa=0.38) reflect mNGS’s unique advantage in detecting rare/atypical pathogens, rather than a lack of reliability. The slightly lower fungal detection rate of mNGS (0.875 vs. culture) may be related to the nucleic acid extraction protocol or bioinformatic annotation bias for fungal sequences, which warrants optimization in future mNGS assays for respiratory samples (Fishman, 2019). Notably, mNGS’s positive criteria (≥10 unique reads + clinical relevance) reduce the risk of false positives caused by background microbial contamination, ensuring the clinical validity of detection results.

### 4.2 mNGS guides antimicrobial stewardship and improves survival

Antimicrobial therapy adjustment in 73.3% of the mNGS group is the core link between mNGS detection and improved clinical outcomes. Unlike the control group, which relied on empirical broad-spectrum antibiotics, mNGS allowed for de-escalation of unnecessary antibiotics in over half of patients and targeted escalation for virus/atypical pathogen infections in nearly half. This precision therapy aligns with the core principles of antimicrobial stewardship (Fishman, 2006; Allerberger and Mittermayer, 2008), reducing antibiotic exposure and the risk of resistance, while ensuring effective treatment for the true causative pathogen.

The multivariable adjusted analysis confirms that mNGS is an independent protective factor for 28-day and 90-day survival in non-verbal elderly SAP patients (adjusted HR = 0.32 and 0.41, respectively). Even after accounting for baseline severity (hypoalbuminemia, electrolyte disturbance, NIHSS), mNGS-guided therapy still notably reduces mortality, which is attributed to timely targeted antimicrobial use and reduced complications such as antibiotic-associated diarrhea and secondary infection. The lower invasive mechanical ventilation rate in the mNGS group (40.0% vs.64.7%) further indicates that mNGS-guided therapy effectively controls pulmonary infection and reduces the risk of respiratory failure progression.

### 4.3 mNGS reduces healthcare resource consumption

A key concern with mNGS application is its perceived high cost (Cieplik et al., 2020), but our results show that mNGS notably reduces total hospitalization costs (¥32,450 vs.¥89,310). This cost reduction is mainly driven by three factors: shorter antibiotic duration (14 vs.21 days), lower invasive mechanical ventilation rate (reducing ICU stay and ventilator-related costs), and shorter overall hospitalization (due to improved infection control). These findings challenge the notion that mNGS is a cost-prohibitive technology and demonstrate its potential cost-effectiveness in SAP management—an important consideration for healthcare systems, especially in resource-limited settings (Kubešová, 2014). The shorter antibiotic duration also has long-term public health benefits by reducing the development of antimicrobial resistance (Muller et al., 2017).From a health economics perspective, the net saving observed in this study (≈¥56,860 per patient) should be interpreted as an association rather than a causal cost-effectiveness estimate, given the retrospective design and absence of formal sensitivity analysis. Nevertheless, the magnitude of the cost difference suggests that the upfront investment in mNGS testing is likely offset by downstream reductions in ventilation needs and antibiotic consumption. Future prospective studies should include a prespecified cost-effectiveness analysis using standardized unit cost data.

### 4.4 Study limitations

This study has several limitations that warrant cautious interpretation of the results: (1) Single-center retrospective design and small sample size (n=64): This limits the generalizability of findings and increases the risk of random error; Statistical analysis was primarily descriptive and comparative without propensity score-based sensitivity analyzes or instrumental variable methods (Dall’Ara et al., 2023). While a biostatistician was not formally involved in the study team, the analytical approach remains appropriate for hypothesis generation in a retrospective cohort of this size; (2) Unmeasured confounding factors: Despite PSM, factors such as ICU care quality, nutritional support and physician experience may affect outcomes; (3) mNGS testing by a third-party laboratory: Lack of standardized in-house mNGS protocols may lead to batch effects; (4) No long-term functional outcome assessment: The study only evaluated 90-day survival and did not assess stroke-related functional recovery, a key outcome for stroke patients; (5) Unadjusted hospitalization costs: Raw cost data do not account for inflation or regional medical price differences. Future research should address these limitations with multicenter prospective cohort studies with larger sample sizes, standardized mNGS protocols, and comprehensive outcome assessment (including functional recovery and long-term antibiotic resistance) (Dall’Ara et al., 2023).

### 4.5 Clinical and policy implications

The median mNGS turnaround time (32 hours) in this cohort is substantially shorter than conventional culture (typically 48–72 hours for preliminary results). Although mNGS results were available after empirical antibiotic initiation (median 3 hours post-diagnosis), the 6-hour interval from result reporting to therapy adjustment indicates that mNGS can inform antimicrobial changes within the same day of result availability. This timeline supports the clinical feasibility of mNGS-guided therapy in non-verbal elderly SAP patients, particularly for de-escalation or targeted escalation before culture results become available. However, the observational design does not allow us to determine whether shorter turnaround time directly caused better outcomes, and the reported intervals should not be interpreted as a threshold for clinical decision-making.

### 4.6 Interpretational caution

The observational design of this study precludes causal inference. Associations between mNGS use and improved outcomes may be influenced by residual confounding, including unmeasured differences in attending physician behavior, nursing intensity, or institutional protocols. Therefore, the reported survival and cost benefits should be considered hypothesis-generating rather than definitive.

### 4.7 Conclusion

Metagenomic next-generation sequencing enables more comprehensive and sensitive pathogen detection in non-verbal elderly stroke-associated pneumonia patients, including the identification of viruses and atypical pathogens missed by conventional culture. mNGS-guided antimicrobial therapy is associated with higher 28-day and 90-day survival rates, lower invasive mechanical ventilation needs, shorter antibiotic treatment durations and reduced hospitalization costs in this high-risk subgroup. Given the single-center retrospective design and limited sample size (n=64), these findings should be interpreted as hypothesis-generating rather than confirmatory. Independent validation in larger, multicenter prospective cohorts with standardized mNGS protocols is required before clinical adoption can be broadly recommended. Future research should also focus on the cost-effectiveness of mNGS and the development of SAP-specific clinical guidelines for mNGS application, to optimize the diagnosis and treatment of this severe stroke complication.

## Data Availability

The original contributions presented in the study are included in the article/supplementary material. Further inquiries can be directed to the corresponding author.
